# Demographics and physical and mental health of clients at a sports center with and without exercise addiction

**DOI:** 10.7717/peerj.19002

**Published:** 2025-03-24

**Authors:** Rasmon Kalayasiri, Chayamon Rattanawijarn

**Affiliations:** 1 Department of Psychiatry, Epidemiology of Psychiatric Disorders and Mental Health Research Unit, Faculty of Medicine, Chulalongkorn University, Bangkok, Thailand; 2 Department of Psychiatry, King Chulalongkorn Memorial Hospital, The Thai Red Cross Society, Bangkok, Thailand; 3 Department of Psychiatry, Program in Mental Health, Faculty of Medicine, Chulalongkorn University, Bangkok, Thailand

**Keywords:** Exercise, Exercise addiction, Knee, Osteoarthristis, Well-being, Mental health

## Abstract

**Background:**

While exercise is widely recognized for its health benefits, excessive engagement can lead to exercise addiction (EA), a behavioral condition characterized by compulsive and harmful physical activity. Limited research exists on the demographic, physical, and mental health profiles of individuals with EA. This descriptive cross-sectional study examines demographic factors and related health outcomes among clients at a university sports center with and without EA.

**Methods:**

A total of 386 participants were randomly recruited through convenience sampling at the Chulalongkorn University Sports Center in Bangkok, Thailand. EA, physical well-being, and mental well-being were assessed using the Exercise Addiction Inventory (EAI), the Knee and Osteoarthritis Outcome Score (KOOS), and the Questionnaire for Eudaimonic Well-Being (QEWB), respectively. Demographic and exercise-related variables, psychological well-being, and knee outcomes were compared between participants with and without EA using Chi-square tests. Logistic regression analysis was performed to identify predictors of EA.

**Results:**

The majority of participants were male (55.2%), with a mean age of 27.5 years (SD = 10.9). Among the 386 participants, 322 (83.4%) exhibited partial symptoms, and 35 (9.1%) exhibited full symptoms of EA. Full symptoms of EA were significantly associated with gender, exercise frequency, smoking status, hours spent walking, hours spent in other sports, cognitive focus on metabolism during exercise, and poor knee outcomes (*P* < 0.05). Logistic regression revealed that being male was the sole significant predictor of EA (OR = 2.65, *P* = 0.024).

**Conclusions:**

EA was prevalent among clients at the sports center and was associated with adverse knee outcomes. Being male was identified as a key predictor of EA. Future research should explore additional factors associated with EA and its effects on physical and mental health.

## Introduction

The rapid development of the economy and society has made physical exercise for physical and mental health a part of daily life for many individuals (Scully et al., 1998). Exercise is widely recognized as a cornerstone of physical and mental well-being, promoting cardiovascular health, muscular strength, and emotional resilience and reducing the risk of chronic diseases and psychological disorders such as depression and anxiety. Regular physical activity is strongly associated with improved mood, cognitive functioning, and overall life satisfaction (Warburton & Bredin, 2017). While exercise offers numerous advantages, excessive or compulsive patterns of physical activity can result in significant disadvantages (O’Connor, 2000; Freimuth, Moniz & Kim, 2011). Exercise addiction (EA) is an obsessive and maladaptive pattern of exercise that negatively impacts physical and psychological health (Freimuth, Moniz & Kim, 2011; Allegre et al., 2007; VanLandeghem, Jakobson & Keough, 2019). Key features of EA include loss of control, symptoms of overtraining, exhaustion, sleep disturbances, and withdrawal symptoms such as restlessness, sadness, and irritability (Hausenblas, Schreiber & Smoliga, 2017). Psychological symptoms may include anxiety, feelings of guilt when not exercising, and social withdrawal, while physical symptoms may involve exhaustion, sleeplessness, headache, and poor appetite. Criteria of EA are tolerance, effects of interrupting exercise, the intended effect, loss of time control, reduction of other activities, and continuity (Hausenblas & Downs, 2002b; Bamber, Cockerill & Carroll, 2000).

The prevalence of EA varies across populations and settings. Studies suggest that EA affects 0.3% to 0.5% of the general population and up to 3%–7% of regular exercisers (Mónok et al., 2012; Griffiths, Szabo & Terry, 2005; Griffiths et al., 2015; Szabo & Griffiths, 2007). Among specific groups, such as elite athletes and fitness enthusiasts, prevalence rates can reach as high as 20% (Griffiths et al., 2015; Hausenblas & Downs, 2002a). Individuals with eating disorders are particularly vulnerable, with EA co-occurring in 39% to 48% of these populations (Freimuth, Moniz & Kim, 2011; Klein et al., 2004). Variations in prevalence underscore the importance of examining contextual and demographic factors influencing exercise behaviors. However, the precise effects remain unclear due to differences in definitions of EA, inconsistent assessment methods, and small sample sizes. Additionally, demographics such as gender and age, and cultural norms may influence susceptibility to EA (Griffiths et al., 2015), though findings remain inconsistent. Understanding these association is crucial for identifying individuals at risk and designing comprehensive treatment approaches. Well-being, a central concept in positive psychology, is defined as a state where individuals achieve success during one’s life through the coordinated work of the body (physical well-being), mind, and intellect, which positively affects society in one way or another. Well-being of the mind is formed by the working together of (1) subjective well-being, the hedonic or happiness that arises from the external environment, which relates to physical health, positive and negative emotions, and life satisfaction, and (2) psychological well-being (Bamber, Cockerill & Carroll, 2000) or Eudaimonic well-being (EWB), where happiness arises within a person’s mentality, who acts in accordance with feelings of being, without expecting the results of the following outcome and can be perceived within oneself in the values and needs of each person (Mónok et al., 2012).

Effect of exercise on well-being has been explored previously. On one hand, moderate, regular physical activity is a potent tool for enhancing quality of life and well-being, contributing to better mental health, improved sleep, and higher energy levels (Penedo & Dahn, 2005). On the other hand, excessive or compulsive exercise can lead to physical injuries, social withdrawal, and psychological distress, including feelings of guilt, anxiety, or frustration when unable to exercise (Lichtenstein et al., 2014). This paradox underscores the need to understand the intricate relationship between exercise, well-being, and EA.

Given the widespread promotion of exercise as a health-enhancing behavior, distinguishing between healthy and pathological exercise patterns is critical. In light of the varying prevalence of EA and its potential to influence well-being and quality of life, identifying the factors related to EA can better inform public health strategies, guide diagnostic criteria development, and improve therapeutic outcomes for affected individuals. This study aims to (1) assess the relationship between demographics and behavioral patterns such as diet and exercise habits with EA among clients at a university sports center, and (2) assess the association between EA and physical and psychological well-being. By doing so, it seeks to contribute to a more comprehensive understanding of exercise behaviors and their implications for health and well-being.

## Materials and Methods

### Study population and participants

This study was conducted from November 2019 to January 2020 at the Chulalongkorn University (CU) Sports Centre, which includes indoor sports building 1–2, CU-Sports Complex, and Chulalongkorn Stadium, where there are many physical activities including fitness, running, swimming, badminton, basketball, and volleyball. Convenient sampling was applied. Inclusion criteria were: (1) individuals aged 18–60 who regularly exercised at least once per week, (2) no communication difficulties, (3) Thai nationality, (4) ability to understand Thai and respond to interview questions and (5) willingness and consent to participate. Exclusion criteria included inability to provide information and regular use of addictive substances.

### Sample size calculation

Sample size was calculated using W.G. Cochran’s formula for an unknown population, with a confidence level of 95% and a margin of error of 5%. The calculation formula applied in this study is:

\begin{eqnarray*}n= \frac{P \left( 1-P \right) {z}^{2}}{{e}^{2}} , \end{eqnarray*}

where:

• *n* = sample size
• *P* = the proportion of the population of interest (if unknown, *P* = 0.5)
• *e* = acceptable margin of error (set at 0.05)
• *z* = 1.96, corresponding to a 95% confidence level

Substituting into the formula:

\begin{eqnarray*}n& = \frac{0.5 \left( 1-0.5 \right) 1.9{6}^{2}}{0.0{5}^{2}} \nonumber\\\displaystyle & =384.16\approx 385. \end{eqnarray*}

Thus, the researcher determined the sample size to be 385 individuals.

### Study instrument

• Exercise Addiction Inventory (EAI) (Terry, Szabo & Griffiths, 2004) was translated by Rasmon Kalayasiri into Thai version for the current study with good internal consistency (Cronbach alpha of Thai EAI = 0.72). It consisted of six items and a five-point Likert scale (*i.e.,* 1 = strongly disagree, 2 = disagree, 3 = neither agree nor disagree, 4 = agree, and 5 = strongly agree). If the total scores ranged from 6 to 30 with EAI score ≥ 24, the subjects exhibit full syndrome of EA; when the score varies between 13 and 23, the subjects exhibit partial symptoms of EA; when the score ranges from 6 to 12, the subjects do not have any symptoms of EA. Therefore, the more likely of having EA when they attain a high EAI score.
• Knee and Osteoarthritis Outcome Score (KOOS) was developed by Roos et al. (1998) and translated into Thai version (Chaipinyo, 2009). It was used to assess and follow up on osteoarthritis patients’ symptoms, consisting of five parts including pain, daily life, a movement for exercise, physical activities, and quality of life. It consisted of 41 items and divided into five levels from no symptom (score = 0) to extreme symptoms (score = 4). The total scores of each part were converted to 100%, then were subtracted from 100 to give a final score, with the low score meaning poor outcomes.
• The Questionnaire for Eudaimonic Well-Being (QEWB) was developed by Waterman et al. (2010) and translated into Thai version (Jarukasemthawee, 2015). It consisted of 21 items and six parts including self-discovery, perceived development of one’s best potential, a sense of purpose and meaning in life, investment of significant effort in pursuit of excellence, intense involvement in activities, and enjoyment of activities as personally expressive. It was five-point Likert scales from strongly disagree to strongly agree. The high scores were presented as good well-being.
• A demographic questionnaire was created for the current study. This included age, gender, marital status, education level, occupation, medical history, exercise habits, diet, and substance use history.

The authors have permission to use EAI, KOOS and QEWB from the copyright holders.

### Data collection

Data collection was conducted at the sports center through bulletin board announcements and direct invitations. If participants agreed to join the study, the researcher explained the objectives and details of the questionnaire and requested their cooperation and consent in participating. Participants completed the questionnaire independently, requiring approximately 30 min per session.

### Data management

Data were systematically entered into a database for analysis, with regular checks for accuracy and completeness. Incomplete questionnaires with substantial missing data were discarded. The quantitative data were checked for normal distribution and, if not passed the threshold, were transformed into categorical variables.

### Statistical analyses

Chi-square tests assessed associations between EA and categorical variables. Physical and psychological well-being were entered into the logistic regression model, with variables with *P* < 0.1 in Chi-square tests for controlling for confounders on EA. The data were analyzed by using SPSS version 22.0. Missing data was excluded from the analysis. A *P*-value <0.05 was considered statistically significant.

### Ethical considerations

The researcher explained the objectives, procedures, and benefits of the study to the participants, who consented to participate voluntarily without receiving any direct benefits. The research procedures and methods adhered to the ethical principles of human research, which include the following three principles. (1) Respect for persons: participants were allowed to make their own decisions after the researcher provided complete information about how their data would be used. In this study, participants’ information will not be disclosed, and their data will be kept confidential. Any potential risks involved in the research were transparently communicated. (2) Beneficence: the study was conducted to ensure no harm to participants. Data collection was carried out in a manner that would not damage the reputation of the participants. (3) Justice: participants were selected based on the inclusion criteria without discrimination based on nationality, religion, or socioeconomic status. Risk distribution was fair among participants. Written informed consent was obtained from all participants. This study was approved by the Ethics Committee, the Institutional Review Board (IRB) of the Faculty of Medicine, Chulalongkorn University (COA No. 342/62).

## Results

All data from 386 participants were included as no substantial missing data was observed. The percentage of subjects who had full symptoms of EA (EAI score ≥ 24) was 9.1% (*n* = 35) and the percentage of those who had partial symptoms of EA was 83.4% (*n* = 322). Table 1 summarizes the univariate findings, including demographics, exercise and dietary habits, and EA among the participants at the sports center.

**Table 1:** Demographics, exercise and diet variables, and exercise addiction among clients at a sports center.

Variables	N = 386	%
Age (Years) Mean (SD) = 27.47 (10.95), Min = 18, Max = 60		
≤ 20	113	29.3
21–30	177	45.9
31–40	34	8.8
41–50	37	9.6
51–60	25	6.4
Gender		
Male	213	55.2
Female	173	44.8
BMI (kg/m^2^) Mean (SD) = 23.13 (4.25), Min = 14.9, Max = 42.3		
≤ 18.50	33	8.5
18.51–24.99	180	46.6
25.00–29.99	83	21.5
≥ 30.00	90	23.3
Being student	244	63.2
History of physical illness	77	19.9
History of mental illness	20	5.2
Substance use		
Never	370	95.9
Quit	16	4.1
Tobacco		
Never/Quit	348	90.2
Current smoker	38	9.8
Frequency of exercise (days/week)		
≤ 3	236	61.1
≥ 4	150	38.9
Spent hours for exercise per day		
<1 h	107	27.7
1–3 h	262	67.9
>3 h	17	4.4
Hours of exercise (≥ 31 min)		
Running (*n* = 213)	90	23.3
Walking (*n* = 135)	45	11.7
Swimming (*n* = 53)	31	80.3
Cycling (*n* = 58)	19	49.2
Weight training (*n* = 115)	56	14.5
Basketball (*n* = 51)	29	75.1
Badminton (*n* = 96)	63	16.3
Others (*n* = 86)	47	12.2
Diet controlling	144	37.3
Diet precaution in meals	136	35.2
Having all food groups for health	304	78.8
Avoiding carbohydrates and sugar	171	44.3
Drinking water instead of soft drinks	263	68.1
Metabolism thinking while exercises	201	52.1
Exercise Addiction Inventory (EAI) (Mean (SD))		
Salience	3.64	(0.896)
Conflict	1.85	(1.034)
Mood modification	3.74	(0.994)
Tolerance	3.51	(0.960)
Withdrawal symptoms	2.83	(1.153)
Relapse	3.11	(1.039)

**Notes.**

BMI Body Mass Index kg kilogram m^2^ square meter SD standard deviation Min minimum Max maximum

The mean age of participants was 27.5 years (SD = 10.9), with 213 males (55.2%) and 173 females (44.8%). Nearly half (46.6%) had a normal Body Mass Index (BMI), while 23.3% were categorized as obese (BMI > 30.00). Most of the subjects attending the sports center were students (63.2%), with 80.8% reporting no physical illness and 95.9% never using substances.

In terms of dietary habits, most participants did not control their diet (62.7%), did not avoid high-calorie foods (64.8%), and consumed all food groups for health (78.8%). However, most subjects considered metabolism during exercise (52.1%), drank water instead of soft drinks (68.1%), but did not avoid sugary foods (55.7%).

Regarding exercise patterns, most subjects (67.9%) spent 1–3 h a day with exercise, and 4.4% spent more than 3 h per day. Moreover, the most common types of exercise were running (55.2%), walking (35.0%), and weight training (29.8%), respectively.

Table 2 highlights the associations between EA and demographic, exercise patterns, and dietary habits. EA was associated with gender, frequency of exercise, current smoking, hours of walking, hours of other sports, and metabolism awareness during exercise (*P* <  0.05).

**Table 2:** Demographic, exercise, and diet variables in a sports center clients with and without exercise addiction.

Variables	Exercise addiction					
	No (*n* = 351)		Yes (*n* = 35)		*χ* ^2^	*p*-value
	*n*	%	*n*	%		
Age (Years)						
≤ 20	107	30.5	6	17.1	3.016	0.221
21 –30	157	44.7	20	57.1		
≥ 31	87	24.8	9	25.7		
Gender						
Male	186	53.0	27	77.1	7.502	0.004^**^
Female	165	47.0	8	22.9		
BMI (kg/m^2^)						
≤ 18.50	32	9.1	1	2.9	1.811	0.613
18.51–24.99	163	46.4	17	48.6		
25.00–29.99	74	21.1	9	25.7		
≥ 30.00	82	23.4	8	22.9		
Being student	222	63.2	22	62.9	0.002	0.550
History of physical illness	68	19.4	9	25.7	0.102	0.478
History of mental illness	16	4.6	4	11.4	3.058	0.096
Substance use						
Never	338	96.3	32	91.4	1.898	0.169
Quit	13	3.7	3	8.6		
Tobacco						
Never/Quit	320	91.2	28	80.0	4.473	0.043^*^
Current smoker	31	8.8	7	20.0		
Frequency of exercise (days/week)						
≤ 3	222	63.2	14	40.0	7.240	0.007^**^
≥ 4	129	36.8	21	60.0		
Spent hours for exercise per day						
<1 h	102	29.1	5	14.3	4.511	0.105
1–3 h	235	67.0	27	77.1		
>3 h	14	4.0	3	8.6		
Hours of exercise (≥ 31 min)						
Running (*n* = 213)	79	22.5	11	31.4	0.603	0.437
Walking (*n* = 135)	37	10.5	8	22.9	3.985	0.048^*^
Swimming (*n* = 53)	29	8.3	2	5.7	0.777	0.378
Cycling (*n* = 58)	16	4.6	3	8.6	0.369	0.544
Weight training (*n* = 115)	45	12.8	11	31.4	2.992	0.072
Basketball (*n* = 51)	23	6.6	6	17.1	1.272	0.259
Badminton (*n* = 96)	59	16.8	4	11.4	0.945	0.331
Others (*n* = 86)	39	11.1	8	22.9	4.754	0.029^*^
Diet controlling	130	37.0	14	40.0	0.119	0.730
Diet precaution in meals	121	34.5	15	42.9	0.980	0.322
Having all food groups for health	273	77.8	31	88.6	2.216	0.097
Avoiding carbohydrates and sugar	156	44.4	15	42.9	0.032	0.857
Drinking water instead of soft drinks	236	67.2	27	77.1	1.439	0.230
Metabolism thinking while exercises	177	50.4	24	68.6	4.198	0.040^*^

**Notes.**

* *P* < 0.05

** *P* < 0.01.

EA, Exercise addiction.

Analyzed by Pearson chi-square and Fisher’s exact test.

Table 3 shows an association between EA and psychological (Eudaimonic) and physical (knee outcome) well-being. The results show that EA was associated with poor knee outcome regarding the poor symptoms aspect of the knee outcome (*P* < 0.05). Psychological well-being and other aspects of knee outcomes including pain, daily activities, movement, and quality of life were not associated with EA (*P* > 0.05). Logistic regression analysis showed that being male was the only predictor of EA (OR = 2.65, *P* < 0.024), as shown in Table 4.

**Table 3:** Psychological (Eudaimonic) and physical (knee outcome) well-being in the sport center clients with and without exercise addiction.

Variables	Exercise addiction					
	No (*n* = 351)		Yes (*n* = 35)		*χ* ^**2**^	*p*-value
	n	%	n	%		
Psychological well-being (Eudaimonic)						
Poor mental health	36	10.3	5	14.3	0.569	0.752
Physical well-being (knee outcome)						
Poor symptoms	10	2.8	4	11.4	6.736	0.034^*^
Poor pain	31	8.8	6	17.1	2.537	0.203
Poor daily activities	18	5.1	3	8.6	0.733	0.294
Poor movement	87	24.8	9	25.7	0.015	0.522
Poor quality of life	105	29.9	14	40.0	1.518	0.149

**Notes.**

* *P* < 0.05.

EA, Exercise addiction.

Analyzed by Pearson chi-square and Fisher’s exact test.

**Table 4:** Predictors of exercise addiction (EA) by logistic regression analysis.

Variables	B	S.E.	O.R.	*p*-value	95% CI	
					Lower	Upper
Being male	0.974	0.432	2.65	0.024^*^	1.135	6.176
Frequency of exercise >3 per week	0.647	0.386	1.91	0.094	0.895	4.071
Metabolism thinking while exercise	0.745	0.401	2.11	0.063	0.960	4.626
Psychological well-being	0.027	0.507	1.03	0.958	0.380	2.775
Physical well-being	0.46	0.382	1.41	0.365	0.668	2.991

**Notes.**

* *P* < 0.05.

## Discussion

This study explored the prevalence and determinants of EA among regular exercisers at a sports center. The prevalence of EA (9.1%) was notably higher than the 3% reported in prior research conducted among gym clients (Mónok et al., 2012), indicating potential contextual or methodological differences. EA was associated with higher frequency and longer exercise durations, and specific activities such as walking and other sports. The results were consistent with the study of Garman et al. (2004), who found that students who worked out more than 360 min per week had a high level of EA . However, it is crucial to distinguish between frequent exercisers with healthy habits and those meeting clinical criteria for EA.

The study revealed a potential relationship between dietary habits and EA. Participants with EA were more likely to consume all food groups for health, possibly reflecting a heightened focus on health. The subjects who thought about calories burning during exercise had EA symptoms higher than those who never think of calories burning. The result may be associated with weight control that was facilitating EA. This aligns with literature suggesting that disordered eating and EA often coexist, with excessive exercise serving as a compensatory behavior for calorie intake. A previous study suggests that the eating behavior should go to the extreme, such as Rocks et al. (2017) found that the hour of physical activities and disordered eating were associated with a high level of EA in students. EA had symptoms in common and high comorbidity with eating disorders (Weinstein & Weinstein, 2014). De Coverley Veale (1987) said that the difference between primary and secondary EA is that primary EA occurs in the absence of an eating disorder. Any weight loss is secondary to calories burned or if there is dieting, it occurs solely for the purpose of improving performance. Sociocultural norms emphasizing fitness and body aesthetics may further exacerbate this association. Individuals may view exercise as a coping mechanism to achieve idealized body standards of the sociocultural norms. Sociocultural factors, including societal pressures to maintain a fit body type, and the extreme fitness regimens and types may also play a role in fostering EA. The interplay between EA and eating patterns may create a reinforcing cycle, where exercise is used to compensate for disordered eating patterns.

Interestingly, participants who were current smokers had a higher prevalence of EA compared to non-smokers or former smokers, contradicting some studies that report lower EA rates among smokers. For example, Szabo et al. (2018) found that subjects with a higher risk of EA had a lower level of prevalence of smokers than those without. This discrepancy warrants further investigation into the interplay between smoking and EA in different populations.

EA was associated with poor knee outcomes, specifically in terms of symptoms, though other aspects of physical well-being were not significantly affected. The absence of a broader impact on physical or psychological well-being suggests that the implications of EA might vary based on the specific context of exercise. Our results were inconsistent with Ersöz (2017), that showed higher psychological well-being levels when the students worked out in advanced levels of exercise. However, the result partially aligns with studies reporting physical injuries and overuse conditions among individuals with EA. A recent study found association between frequent physical injuries and EA among runners in Croatia (Zivcic Tomic et al., 2023).

Gender differences emerged as a key finding, with males more likely to exhibit EA symptoms. The logistic regression results showed that being male was the only predictor for EA in our study. This could reflect differing motivations for exercise across genders, such as performance-driven goals in men *versus* body image concerns in women. In addition, the interpretation of the EA scale was different across genders (Griffiths et al., 2015). There are potential psychosocial determinants of exercising that could have confounded the associations observed across genders. Gender may moderate the relationship between well-being and exercise addiction by influencing how psychological and physical well-being affect the behaviors. The sociocultural and psychological determinants of these differences warrant further exploration.

Despite its contributions, this study has limitations. The high prevalence of partial EA symptoms (83.4%) suggests potential over-sensitivity of the EA assessment tool. Additionally, the study’s cross-sectional design and reliance on self-reported data may introduce biases and limit causal inferences. Finally, the absence of measures for other psychiatric conditions, such as eating disorders, restricts a comprehensive understanding of EA’s comorbidities. Future investigators should use more objective measurements to verify the physical and mental well-being, to measure these complex issues, and to include other psychiatric disorders in the future studies.

In Thailand nowadays, government organizations promote activities of regular exercises for health and well-being. Thai Health Promotion Foundation (2015), which supports exercise and sports events, plans to increase the physical activity of Thai people aged 11 years and older to at least 80 percent by 2021. However, the understanding of EA among Thai people should be more educated and explored generally.

## Conclusions

The study found that gender, considering metabolism during workouts, the frequency of workouts and the number of hours of walking was associated with EA among clients at a sports center. Being male was the only predictor for EA. EA was associated with physical well-being such as poor knee outcome, but not associated with psychological well-being. The limitation of this study was a cross-sectional descriptive in a specific area in which the result may not be represented to all populations. However, future studies should clarify the relationship between EA and other mental health problems, also understanding EA or overtraining conditions in general.

##  Supplemental Information

10.7717/peerj.19002/supp-1 Supplemental Information 1 The dataset of demographics, exercise, physical outcome and quality of life of clients at the sport centre

10.7717/peerj.19002/supp-2 Supplemental Information 2 STROBE checklist

## Additional Information and Declarations

### Data Availability

The following information was supplied regarding data availability: The raw measurements are available in the Supplementary File.
